# Deep Eutectic Solvents as More Sustainable Dispersion Media for Beeswax Coatings from Cultural Heritage: Experimental Insights and Theoretical Modeling

**DOI:** 10.1002/asia.202500673

**Published:** 2025-07-08

**Authors:** Andrea Macchia, Raffaella Mancuso, Romina Strangis, Irene Angela Colasanti, Mariangela Novello, Mauro Francesco La Russa, Bartolo Gabriele, Roberto Beneduci

**Affiliations:** ^1^ YOCOCU (YOuth in COnservation of CUltural Heritage) Via T. Tasso 108 Rome 00185 Italy; ^2^ Department of Biology, Ecology and Earth Science (DiBEST) University of Calabria Arcavacata di Rende CS 87036 Italy; ^3^ Laboratory of Industrial and Synthetic Organic Chemistry (LISOC), Department of Chemistry and Chemical Technologies University of Calabria Via Pietro Bucci 12/C Arcavacata di Rende CS 87036 Italy; ^4^ Sciences and Chemical Technologies Department Tor Vergata University Rome via della Ricerca 7 Scientifica 1 Roma 00133 Italy; ^5^ Department of Physics University of Calabria, and INFN gruppo coll. Cosenza via P. Bucci 31/C Arcavacata di Rende CS 87036 Italy

**Keywords:** Beeswax, Cultural heritage, Green solvents, Hydrophobic natural deep eutectic solvents, Kinetic modeling, Regression models

## Abstract

Deep eutectic solvents (DESs) are gaining attention as sustainable alternatives to traditional organic solvents, particularly for applications involving nonpolar substances. In this study, the dispersion behavior of beeswax in a selection of hydrophobic natural deep eutectic solvents (hNADESs) was investigated using both theoretical and experimental approaches. The compatibility between each DES and beeswax was first assessed through Hansen solubility parameters and RED (relative energy difference) values. Based on these results, a subset of DESs was selected for kinetic testing. Turbidimetric and gravimetric measurements were carried out over time to monitor the dispersion process. The experimental data were then fitted using a simplified Hill‐type kinetic model, which successfully described the nonlinear, saturation‐limited behavior observed in the systems. DES 8, composed of dodecanoic and decanoic acids, showed the highest dispersion capacity, while DES 6 (tymol and L‐(−)‐menthol) exhibited moderate performance. DES 12, containing choline chloride and diglycolic acid, showed negligible activity. The findings highlight the relevance of combining solubility theory with kinetic modeling to evaluate hNADES performance. The proposed approach offers a useful tool for predicting and optimizing dispersion processes in structured, low‐toxicity solvents.

## Graphical abstract

The work reports a combined theoretical modeling and experimental study aimed at evaluating the appropriate use of deep eutectic solvents (DESs) as green dispersion media for beewax removal from cultural heritage surfaces.

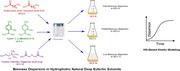

## 1 Introduction

Deep eutectic solvents (DESs) are emerging as promising, environmentally sustainable alternatives to conventional organic solvents due to their low toxicity, high biodegradability, and ease of preparation from inexpensive, naturally derived components.^[^ ^1^, ^2^, ^3^ ^]^ These solvents, formed by mixing hydrogen bond donors (HBDs) and hydrogen bond acceptors (HBAs), exhibit melting points significantly lower than those of their individual constituents, remaining liquid at or near room temperature.^[^ ^4^, ^5^ ^]^ The physicochemical tunability of DESs enables the solubilization of both polar and nonpolar compounds, thereby broadening their application in fields such as extraction, materials science, and green chemistry.^[^ ^6^, ^7^ ^]^ Among the various classes of DESs, hydrophobic DESs (hDESs) have garnered increasing interest for their ability to dissolve nonpolar substances while maintaining immiscibility with water.^[^ ^8^, ^9^, ^10^, ^11^, ^12^ ^]^ This property makes hDESs particularly suitable for applications in the cultural heritage sector, where they have been proposed as safer, greener alternatives to traditional solvents for the removal of aged, hydrophobic coatings from sensitive substrates.^[^ ^13^ ^]^ Recent developments include the incorporation of DESs into polymeric gels for localized cleaning interventions demonstrating their adaptability to complex conservation challenges.^[^ ^14^, ^15^ ^]^

We recently reported the use of hDESs for the removal of nonpolar coatings from works of art, including beeswax, a widely used hydrophobic coating in the cultural heritage conservation field.^[^ ^13^ ^]^ In particular, we found that fatty acid‐based hDESs with longer alkyl chains (such as dodecanoic acid/decanoic acid 1:2 or l‐(‒)‐menthol/octanoic acid 1:1) were efficient in beeswax removal due to their affinity to the structure of beeswax’ constituents.^[^ ^13^ ^]^ In the present study, we have theoretically evaluated the solubility and dispersion kinetics of beeswax within a series of hydrophobic natural DES (hNADES) formulations. Beeswax's complex composition and limited solubility in aqueous or polar solvents make its removal particularly challenging.^[^ ^16^ ^]^ The objective is to identify hNADES systems capable of dispersing beeswax under mild, noninvasive conditions, thereby supporting selective and controlled cleaning procedures. The methodology integrates theoretical solubility screening using Hansen solubility parameters (HSP), Teas diagrams, and the relative energy difference (RED) criterion^[^ ^17^, ^18^, ^19^, ^20^, ^21^, ^22^, ^23^, ^24^ ^]^ with time‐resolved experimental analysis via turbidimetry and gravimetry,^[^ ^24^, ^25^ ^]^ while previous studies have described wax or lipid dispersion through empirical models (e.g., zero‐ or first‐order kinetics) or by applying simplified versions of the Noyes–Whitney or Higuchi equations, such approaches often fail to capture the sigmoidal trends and concentration‐dependent behavior observed in structured solvents.^[^ ^24^, ^25^, ^26^, ^27^, ^28^ ^]^ Moreover, purely Fickian models assume constant diffusion coefficients and linear concentration gradients, which may not reflect real behavior in viscous or semi‐structured environments such as DESs.^[^ ^25^, ^29^ ^]^ To overcome these limitations, this work adopts a kinetic perspective, modeling the time evolution of wax dispersion through a Hill‐type regression function with a fixed shape factor. This choice is motivated by the need to describe dispersion behavior in structured solvents where transport is often hindered by viscosity, molecular interactions, and steric effects. Previous studies have demonstrated that the Hill equation provides an effective fit for saturation‐limited and sigmoidal dispersion processes, particularly in non‐Fickian systems such as lipophilic compound diffusion in supercritical fluids or colloidal suspensions.^[^ ^30^, ^31^ ^]^ The use of a fixed shape parameter simplifies the fitting procedure while preserving the essential curvature of the kinetic profile. By combining predictive solubility models with empirical kinetic data, supported by experimental data, this study aims to establish a robust framework for selecting and optimizing hNADES formulations for conservation purposes. The results contribute to a deeper understanding of DES–wax interactions and offer a reproducible, quantitative approach for evaluating the performance of green solvents in the removal of hydrophobic materials from heritage surfaces.

## 2 Results and Discussion

### 2.1 Hydrophobic Natural Deep Eutectic Solvents (hNADESs)

Twelve natural deep eutectic solvents (NADESs) were synthesized and characterized for this study (Table 1). The selection was guided by a structural analysis of HBDs and HBAs, with attention to polarity balance and functional group distribution. Emphasis was placed on optimizing interactions with hydrophobic substrates such as natural and microcrystalline waxes. DESs containing thymol and medium‐chain fatty acids (e.g., decanoic acid) combine aromatic and aliphatic hydrophobic moieties with polar functionalities (–OH, –CO_2_H), offering amphiphilic properties favorable for wax solubilization. Systems based on l‐(−)‐menthol and long‐chain carboxylic acids (e.g., dodecanoic acid) exhibit enhanced hydrophobicity, suitable for dispersing high‐molecular‐weight waxes. For comparison, highly polar DESs based on choline chloride and organic acids (e.g., tannic acid, diglycolic acid) were included to evaluate incompatibility with nonpolar substrates. All DES used and present in Table 1 were prepared by the Laboratory of Industrial and synthetic organic chemistry (LISOC) within the Department of Chemistry and Chemical Technologies at the University of Calabria. For the synthesis of DESs, thymol, l‐(−)‐menthol, tetrabutylammonium bromide, betaine, choline chloride, tannic acid, diglycolic acid, octanoic acid, decanoic acid, and dodecanoic acid were purchased from Merck.^[^ ^32^, ^33^, ^34^, ^35^, ^36^, ^37^, ^38^, ^39^, ^40^, ^41^ ^]^ Components 1 and 2, at the proper molar ratio (as shown in Table 1), were mixed and gently heated at 60 °C–100 °C on a magnetic stirrer hotplate (VELP AREX Digital Pro) under constant stirring until homogeneous and transparent liquids were obtained, in times ranging from 1 to 3 h. Molar ratios were precisely measured by mass using an analytical balance (Mettler Toledo XPR105, ±0.01 mg). Synthesized hNADESs were stored in airtight amber vials under nitrogen atmosphere to prevent moisture uptake. For simplicity in the text the hNADESs will be called as DESs.

**Table 1:** Composition of hNADESs tested in the experimentation.

DES name	Component 1	Component 2	Molar ratio
**DES 1**	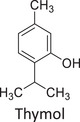 Thymol	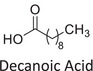 Decanoic acid	1:1^[^ ^32^ ^]^
**DES 2**	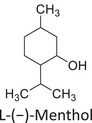 L‐(−)‐Menthol	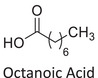 Octanoic acid	1:1^[^ ^33^ ^]^
**DES 3**	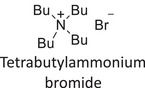 Tetrabutylammonium bromide	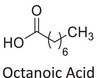 Octanoic acid	1:1^[^ ^34^ ^]^
**DES 4**	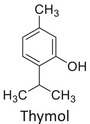 Thymol	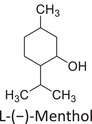 L‐(−)‐Menthol	1:2^[^ ^35^ ^]^
**DES 5**	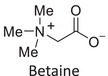 Betaine	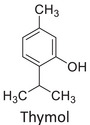 Thymol	1:3^[^ ^36^ ^]^
**DES 6**	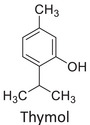 Thymol	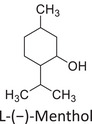 L‐(−)‐Menthol	1:1^[^ ^35^ ^]^
**DES 7**	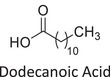 Dodecanoic acid	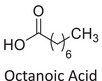 Octanoic acid	1:3^[^ ^37^ ^]^
**DES 8**	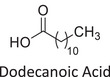 Dodecanoic acid	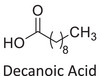 Decanoic acid	1:2^[^ ^37^ ^]^
**DES 9**	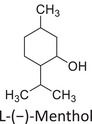 L‐(−)‐Menthol	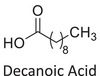 Decanoic acid	1:1^[^ ^38^ ^]^
**DES 10**	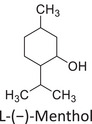 L‐(−)‐Menthol	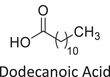 Dodecanoic acid	3:1^[^ ^39^ ^]^
**DES 11**	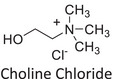 Choline chloride	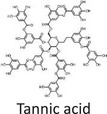 Tannic acid	20:1^[^ ^40^ ^]^
**DES 12**	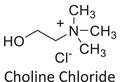 Choline chloride	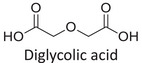 Diglycolic acid	1:2^[^ ^41^ ^]^

### 2.2 Hansen Solubility Parameter Calculations

The solubility behavior of each DES was evaluated using Hansen solubility parameters (HSP), determined via the Hoftyzer–Van Krevelen group contribution method.^[^ ^42^ ^]^ The partial solubility parameters‐dispersion (*δ*_d), polar (*δ*_p), and hydrogen bonding (*δ*_h)‐were calculated from known functional group constants using the following equations:

$$\delta_{d}=\frac{\Sigma F_{\mathrm{di}}}{V}$$

$$\delta_{p}=\frac{\sqrt{\sum F_{\mathrm{pi}}^{2}}}{V}$$

$$\delta_{h}=\sqrt{\frac{\sum E_{\mathrm{hi}}}{V}}$$

The variables *F*_di_ and *F*_pi_ represent the dispersive and polar components, respectively, based on the molar attraction constant, F, proposed by Small,^[^ ^43^ ^]^ while *E*_hi_ denotes the hydrogen bonding energy. The term *V* refers to the molar volume of the molecule. In the present study, we followed Hoftyzer–Van Krevelen method^[^ ^42^ ^]^ and used the specific values of *F*_di_, *F*_pi_, and *E*_hi_ given in Ref. [42], Table 7.10, page 215.

Then, the molar volume of the constituents in the DES mixture is calculated by means of the following equations:

$$\varphi_{i=\frac{x_{i}v_{i}}{\Sigma_{j}x_{i}v_{j}}}$$

*x*_i_ and *x*_j_ represent the molar fraction of components i and j, respectively, while *v*_i_ and *v*_j_ are the molar volume of each component.and the overall DES parameters as:

$$\delta_{d}^{M}=\delta_{d^{1}}^{s1}\varphi_{1}+\delta_{d_{2}}^{s_{2}}\varphi_{2}$$

$$\delta_{p}^{M}=\delta_{p^{1}}^{s1}\varphi_{1}+\delta_{p_{2}}^{s_{2}}\varphi_{2}$$

$$\delta_{h}^{M}=\delta_{h^{1}}^{s1}\varphi_{1}+\delta_{h_{2}}^{s_{2}}\varphi_{2}$$

The results of the calculation are reported in Table 2. Hansen solubility parameters related to wax were recovered using the parameters of white spirit, solvent commonly used to remove beewax.

**Table 2:** Hansen and Teas parameters of tested DESs.

	Hansen parameters			Teas parameters		
ID	*δ*_d_	*δ*_p_	*δ*_h_	*F*_d_	*F*_p_	*F*_h_
**DES 1**	15.0	2.3	7.8	60	9	31
**DES 2**	14.6	2.4	8.0	59	9	32
**DES 3**	17.9	2.7	4.4	72	11	18
**DES 4**	14.2	2.4	8.4	57	9	34
**DES 5**	14.2	2.3	8.5	57	9	34
**DES 6**	13.7	3.1	8.2	55	13	33
**DES 7**	15.5	2.3	7.2	62	9	29
**DES 8**	16.0	2.1	7.0	64	8	28
**DES 9**	15.0	2.2	7.8	60	9	31
**DES 10**	14.8	2.2	8.0	59	9	32
**DES 11**	11.4	3.8	9.8	46	15	39
**DES 12**	11.0	4.8	9.3	44	19	37

As a second method, we used Teas chart^[^ ^44^, ^45^, ^46^, ^47^, ^48^, ^49^ ^]^ in order to estimate the solubility parameters of the different DESs. Teas chart is a valuable tool widely used by restorers in the conservation of cultural heritage. It is derived from Hansen's solubility parameters and is utilized to identify the solubility regions of solvents, providing essential information for the cleaning and preservation of artifacts, based on the following equations:

The calculated Hansen parameters are used to derive the values of F_d_, F_p_, and F_h_ (Teas diagram):

$$F_{d}=\frac{\delta_{d}}{\delta_{d}+\delta_{p}+\delta_{h}}x100$$

$$F_{p}=\frac{\delta_{p}}{\delta_{d}+\delta_{p}+\delta_{h}}x100$$

$$F_{h}=\frac{\delta_{h}}{\delta_{d}+\delta_{p}+\delta_{h}}x100$$

$$F_{d}+F_{p}+F_{h}=100$$

**Figure 1:**
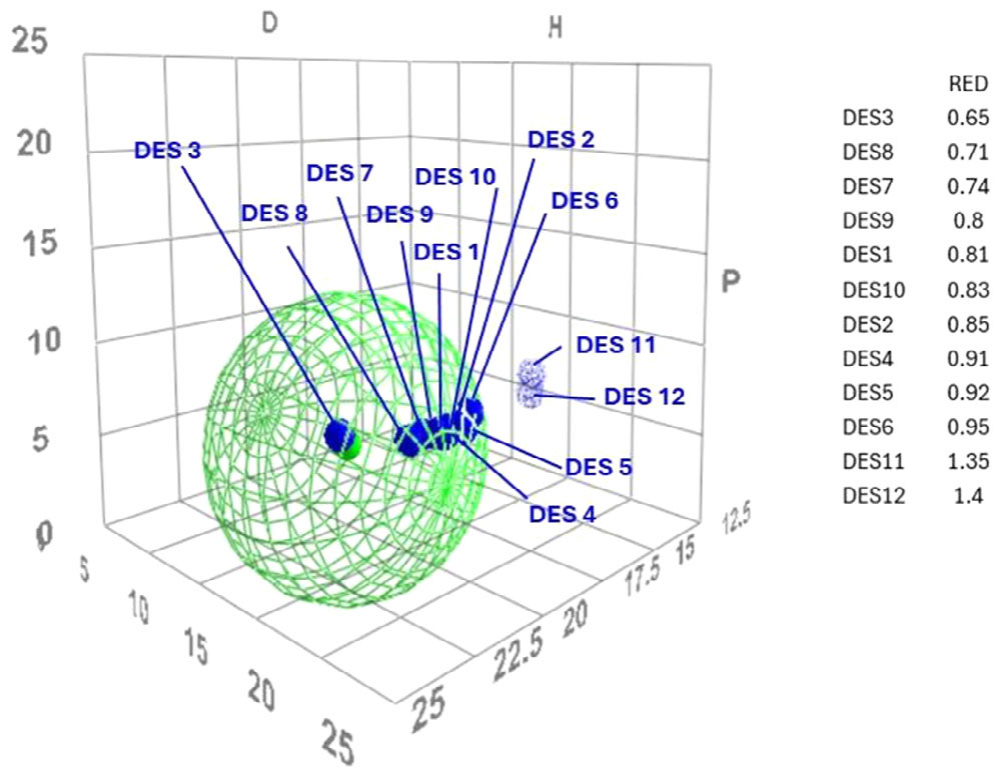
Spatial distribution of the DESs in Hansen solubility space relative to beeswax (green sphere). The size of the sphere represents the solubility radius of beeswax, and the proximity of each DES (blue points) to the center correlates with increasing theoretical compatibility, with RED values reported in label.

This triangular diagram simplifies Hansen's parameters by representing solvents according to three coordinates, allowing for a more accessible interpretation of their solubility characteristics. Solvents that are represented by points in close proximity on the chart tend to exhibit similar solvent power. The results are represented in the Teas chart (Figure 1).

Solubility compatibility between each DES and beeswax was estimated using the RED parameter. In Hansen space the solubility parameter, denoted by *R_a_*, determines the distance between a solute and a solvent, or more generally, between two substances being studied. The *R_a_* value is calculated using the following formula:

$$R_{a}^{2}=4{\left(\delta_{d1}-\delta_{d2}\right)}^{2}+{\left(\delta_{p1}-\;\delta_{p2}\right)}^{2}+{\left(\delta_{\mathrm{hb}1}-\delta_{\mathrm{hb}2}\right)}^{2}$$

where the subscripts 1 and 2 refer to the solute (beeswax) and the solvent (DES), respectively. A lower *R*_a_ value indicates a higher compatibility between the solute and the solvent, suggesting that the solvent is well‐suited for dissolving the solute. To further assess the efficiency of the DESs, the RED parameter was determined using the equation:

$$\mathrm{RED}=\frac{R_{a}}{R_{0}}$$

where *R*_0_ represents the maximum value of *R*_a_ at which a solvent is still considered effective. A RED value close to 0 suggests a perfect match between the solute and the solvent, RED values less than 1 indicate strong cohesion and high solubilization potential, while higher RED values suggest lower efficacy of the solvent for the specific solute. The results of the calculations are reported in Table 3.

**Table 3:** Regression statistics for turbidity and gravimetric measurements of **DES 8** [dodecanoic acid/decanoic acid (1:2)] and **DES 6** [thymol/l‐(−)‐menthol (1:1)].

	DES 8		DES 6	
	Turbidity	Gravimetric	Turbidity	Gravimetric
*R* ^2^	0.999	0.988	0.973	0.996
*aR* ^2^	0.998	0.982	0.957	0.994
*StdErr* (*SE*)	27.577	0.19	20.59	0.04
**P**	0.000000031	0.0000015	0.000046	0.000000097
**AIC**	77.4	−2.1	72.8	−26.978
**BIC**	77.6	−1.9	72.96	−26.82
**F**	952	258.6	80	648.68
**DoF**	6	6	6	6
**AICc**	79.87	0.26	75.2	−24.58

This analysis provides a theoretical assessment of the solubilizing power of the DESs to be tested experimentally (see Section 3).

### 2.3 Experimental Study of the Dispersion Process

Following the theoretical pre‐screening based on Hansen solubility parameters and RED values, three hNADES formulations [DES 8: dodecanoic acid/decanoic acid (1:2); DES 6: thymol/l‐(−)‐menthol (1:1); DES 12: choline chloride/diglycolic acid (1:2)] were selected for experimental validation. To investigate the kinetics of beeswax dispersion in the selected hDESs, a standardized experimental protocol was applied.

Precisely 5.00 ± 0.01 g of finely ground, refined beeswax (Sigma‐Aldrich, W8000) was weighed using an analytical balance (Sartorius Cubis II, readability 0.01 mg) and placed in 10 mL borosilicate glass test tubes. Each tube was then filled with 5 mL of the corresponding hDES. The tubes were hermetically sealed with PTFE‐lined screw caps to prevent evaporation and contamination during the experiment. Samples were incubated in a thermostatically controlled shaking bath (Julabo SW23) at 30 ± 2 °C. Continuous orbital stirring at 150 rpm was applied using an IKA KS 4000 i control system to ensure uniform mixing throughout the process. Dispersion evolution was monitored at predetermined time intervals: 30, 60, 120, 360, 480, and 720 min. For each time point, an individual test tube was dedicated. At the scheduled time, the tube was removed, and the upper phase containing the DES with dispersed wax was carefully separated from the solid residue, avoiding disturbance of the sedimented wax layer. Turbidity of the withdrawn phase was immediately measured using a calibrated Lovibond TB 211 IR turbidimeter, providing a rapid and nondestructive estimate of the wax dispersed in colloidal or micellar form. The remaining solid wax was recovered from the tube and weighed using the same analytical balance. In addition, the height of the residual wax layer was measured. The difference between the initial and final wax mass was used to calculate the dispersed fraction, thus complementing the turbidity measurements. As an additional metric, the thickness of the residual wax adhering to the bottom of each test tube was recorded using a Mitutoyo Surftest SJ‐500 contact profilometer. This independent measurement provided further insight into the dissolution process. All experiments were performed in triplicate. Mean values and standard deviations were calculated for each dataset to ensure statistical validity. Curve fitting of turbidity and gravimetric data was performed using the “Mycurvefitting” software.

### 2.4 Solubility Parameter Calculations and Compatibility Predictions

The Hansen solubility parameters (*δ*_d_, *δ*_p_, *δ*_h_) and the TEAS contributions (*F*_d_, *F*_p_, *F*_h_) for each hDES are reported in Table 2. The dispersive components (*δ*_d_) range from 11.0 (DES 12) to 17.9 (DES 3), while the polar and hydrogen‐bonding components span from 2.1 to 4.8 and from 4.4 to 9.8 MPa 1/2, respectively.

Figure 1 reports the solubility values between beeswax and the twelve DESs selected for the study. The lowest RED values among the tested DESs were observed for DES 3 (0.65), DES 8 (0.71), DES 7 (0.74), and DES 9 (0.80). These are followed by DES 1 (0.81), DES 10 (0.83), DES 2 (0.85), DES 4 (0.91), DES 5 (0.92), and DES 6 (0.95). The highest RED values, exceeding the compatibility threshold, were recorded for DES 11 (1.35) and DES 12 (1.40).

DESs with the lowest RED values are characterized by the presence of medium‐ or long‐chain carboxylic acids (DES 7, DES 8, DES 9, DES 10), nonpolar terpenic alcohols such as l‐(−)‐menthol (DES 9, DES 10), or bulky alkyl cations, as in the case of tetrabutylammonium (DES 3). These components exhibit low polarity and reduced hydrogen‐bonding capability, properties that favor affinity with nonpolar substances like beeswax.

DES 1, composed of thymol and decanoic acid, and DES 2, consisting of menthol and octanoic acid, show moderate RED values, consistent with the amphiphilic nature of these systems. DESs based on thymol and menthol combinations (DES 4, DES 6), or on betaine (DES 5), feature a higher content of hydrogen‐bond donor groups (–OH, –CO_2_H, –NH), leading to increased polarity and therefore lower compatibility compared to purely nonpolar systems.

The highest RED values were observed for DES 11 and DES 12, which contain choline chloride combined with strongly polar organic acids such as tannic acid or diglycolic acid. In both cases, the predominance of hydrophilic polar groups (phenolic, multiple carboxylic, and ether functionalities) results in a high hydrogen‐bonding component (*δ*_h), markedly different from the solubility profile of beeswax.

**Figure 2:**
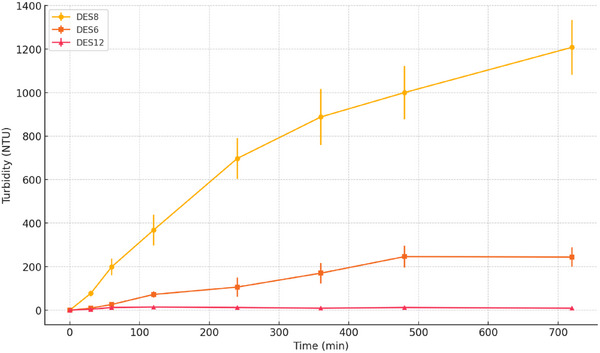
Trend of turbidity (NTU) over time (minutes) for the tested DESs [**DES 8**: dodecanoic acid/decanoic acid (1:2); **DES 6**: thymol/l‐(−)‐menthol (1:1); **DES 12**: choline chloride/diglycolic acid (1:2)].

For the subsequent experimental phase, three DESs were selected based on their calculated RED values with respect to beeswax: DES 8, DES 6, and DES 12. DES 8 was chosen as a representative of high theoretical compatibility. DES 6 was selected as a partially compatible system, close to the threshold value of RED = 1. DES 12 showing the highest RED value, used as a reference for low compatibility. Figure 2 reports the time‐dependent turbidity values (NTU) measured for the three selected DESs (DES 8, DES 6, DES 12) over a 720 min interval. The turbidity, and consequently the degree of wax dispersion, increases over time for both DES 8 and DES 6, while DES 12 shows no significant increase and appears unable to disperse the wax under the tested conditions. As expected, DES 8 exhibits the highest turbidity values throughout the experiment. However, the increase is not constant, with a progressive slowdown observed in the final stages. In contrast, DES6 shows a more linear trend, but with an even more defined plateau region than DE8, with turbidity rising steadily over time, though with markedly lower values than those recorded for DES 8.

**Figure 3:**
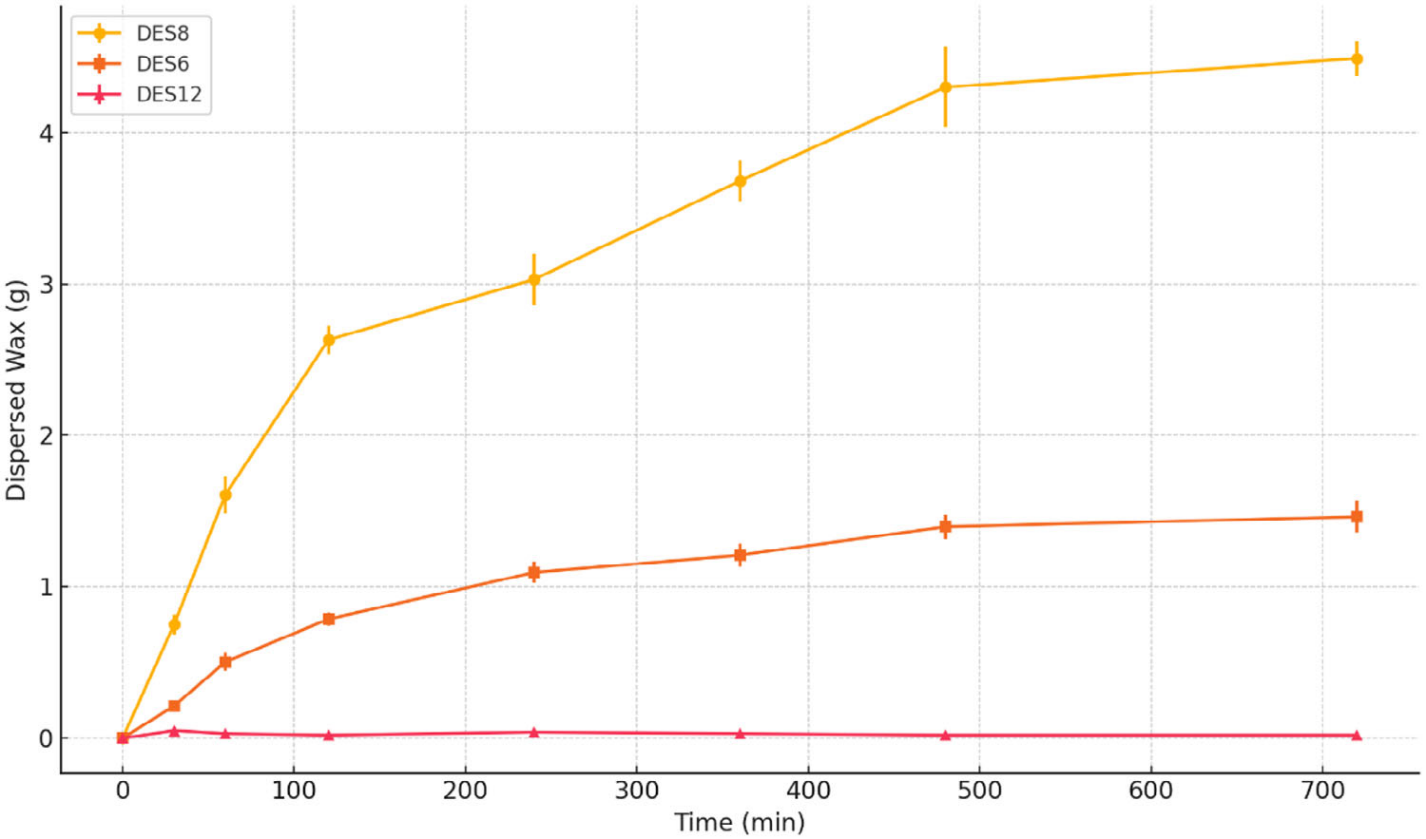
Trend of amount of dispersed wax over time (minutes) for the tested DESs [**DES 8**: dodecanoic acid/decanoic acid (1:2); **DES 6**: thymol/l‐(−)‐menthol (1:1); **DES 12**: choline chloride/diglycolic acid (1:2)].

Figure 3 shows the amount of wax dispersed over time in DES 8, DES 6, and DES 12, measured gravimetrically. DES 8 leads to the highest dispersion, reaching approximately 4.5 g at 720 min, with a marked increase in the first 360 min followed by a slower growth. DES 6 exhibits an intermediate behavior, with steady dispersion reaching about 1.46 g at the final time point. DES 12 shows minimal dispersion throughout the experiment, with values remaining near 0.02 g, confirming a negligible interaction with beeswax under the tested conditions.

### 2.5 Fitting of the Data and Regression Model

Experimental data obtained from turbidity and gravimetric measurements were fitted using a Hill‐type equation with a shape parameter equal to 1 (see Equation 1 below). This two parameters model, formally equivalent to the Michaelis–Menten equation, was applied to describe the time evolution of wax dispersion in DES 8 and DES 6. DES 12 was excluded from the regression analysis due to the negligible solubilization of beeswax, as shown by the lack of measurable variation in data.

(1) $$f\left(t\right)=a\left(1-\frac{1}{1+\frac{t}{b}}\right)$$

**Figure 4:**
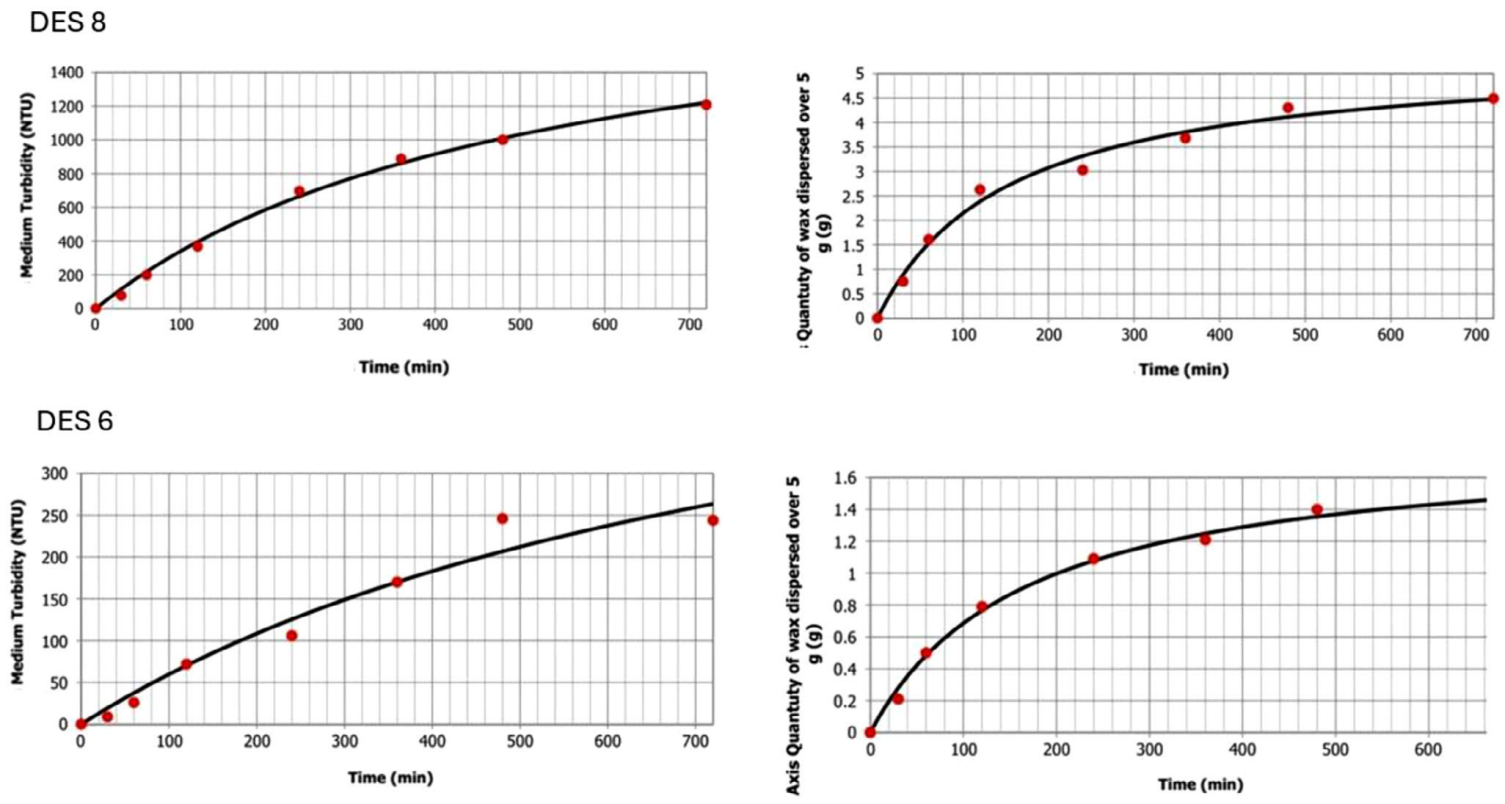
Fitting of experimental data using the Hill‐type equation (shape parameter = 1) for **DES 8** [dodecanoic acid/decanoic acid (1:2)] (top) and **DES 6** [thymol/l‐(−)‐menthol (1:1)] (bottom). The fittings have been realized by means of the software MyCurveFit (https://mycurvefit.com/).

Fitting of experimental data using the Hill‐type equation for DES 6 and DES 8 are reported in the Figure 4. For each system, the fitting captures both the rapid initial phase and the slower approach to equilibrium observed for DES 8, as well as the more gradual progression seen in DES 6. In all cases, the model provides a coherent representation of the dispersion process, supporting its use as a predictive tool for quantifying the extent and rate of solubilization.

Table 3 summarizes the main statistical parameters for the regression: determination coefficient (*R* ^2^), adjusted *R* ^2^ (a*R* ^2^), standard error (SE), *p*‐value, Akaike Information Criterion (AIC), Bayesian Information Criterion (BIC), and F‐statistic. For DES 8, *R* ^2^ values reach 0.999 for turbidity and 0.988 for mass, indicating excellent fit. Similar results are observed for DES 6. According to the table, the model demonstrates high explanatory power, as reflected by the high determination coefficients. However, the values of the standard error (SE) suggest that its predictive accuracy is more limited. For example, in the case of turbidimetric measurements for DES 8, the SE is 27.577, corresponding to a prediction interval of approximately 55.2 NTU. When compared with the actual turbidity values, this translates into relative errors of 14% at *t* = 120 min, 7.8% at *t* = 240 min, and 4.5% at *t* = 720 min. For gravimetric measurements with DES 8, the SE is 0.19, yielding relative errors of 14% at *t* = 120 min, 12.5% at *t* = 240 min, and 8.5% at *t* = 720 min. Comparable trends are observed for DES 6. In the gravimetric dataset, the relative error is 10.1% at *t* = 120 min and 5.5% at *t* = 720 min. For the turbidimetric dataset, the relative error is more pronounced, reaching 57.2% at *t* = 120 min and 16.8% at *t* = 720 min.

### 2.6 Kinetics Model

**Figure 5:**
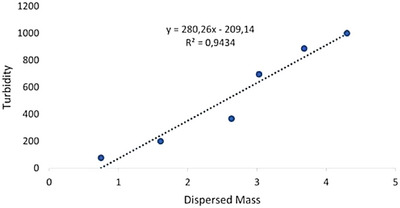
Linear correlation between turbidity and dispersed wax mass in **DES 8** [dodecanoic acid/decanoic acid (1:2)].

The approximately linear relationship between turbidity and dispersed wax mass observed in the data is consistent with the empirical equation proposed by Sutherland^[^ ^50^ ^]^ and suggests that multiple particle scattering effects are negligible, in agreement with the findings of H. H. Kleizen et al.^[^ ^51^ ^]^ The correlation between turbidity and dispersed wax mass in DES8 follows a linear trend with a determination coefficient of *R* ^2^ = 0.9434 (Figure 5). The resulting equation, *y* = 280.26x–209.14, confirms that turbidity increases proportionally with the amount of dispersed wax.

**Figure 6:**
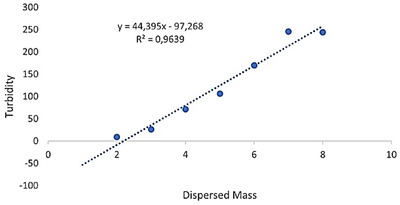
Linear fit between turbidity and dispersed wax mass in **DES 6** [thymol/l‐(−)‐menthol (1:1)].

In the case of DES 6, turbidity shows a strong linear dependence on the amount of dispersed wax, as evidenced by an *R* ^2^ value of 0.9639 (Figure 6). The regression line indicates a stable proportionality between the two variables across the tested range, suggesting that changes in turbidity reliably reflect the progression of the dispersion process.

The experimental dispersion curves for DES8 and DES6—both turbidimetric and gravimetric—exhibited a characteristic behavior described by the Hill function with a shape parameter *c* = 1, corresponding to a non‐autocatalytic process.

To capture this behavior mathematically, we refer to the generalized Hill equation (Equation (2)).^[^ ^30^ ^]^

(2) $$\frac{f\left(t\right)}{a}=1-\frac{1}{1+{\left(\frac{t}{b}\right)}^{c}}$$

This function has been used in several contexts, including autocatalytic reactions, pharmacokinetic–pharmacodynamic modeling, and economics. Its connection to chemical equilibrium theory has also been widely described.^[^ ^52^, ^53^, ^54^ ^]^ The best fit was obtained with *c* = 1, which simplifies Equation (2) to Equation (1):

(1) $$f\left(t\right)=a\left(1-\frac{1}{1+\frac{t}{b}}\right)$$

By using the theory of ordinary differential equations, it is easy to prove that Equation (1) is the solution of the differential Equation (3)(:

(3) $$\frac{df}{dt}=\frac{a}{b}{\left(1-\frac{f}{a}\right)}^{2}$$

from which it can be seen that the constant *a* is the saturation concentration of the wax in the DES. Indeed, when *f*  =  *a*, the derivative $\frac{df}{dt}=0$ so that the concentration is a constant; it cannot increase anymore. Furthermore, by Equation (1) it follows that the coefficient *b* corresponds to the time at which $f=\frac{a}{2}$, that is, the time needed for the DES to be half saturated.

Equation (3) shows that the dispersion rate, $\frac{df}{dt}$, decreases with the square of the saturation level of the DES, ${\left(1-\frac{f}{a}\right)}^{2}$. In other words, the dispersion rate decreases with the increasing of the concentration of the wax in the DES but, at variance with the Noyes and Whitney equation which characterizes the diffusion of a solid substance in its own solution,^[^ ^22^, ^23^ ^]^ such a decrease is quadratic, not linear.

In order to make a comparison between Equation (3) and the Noyes–Whineyequation (Equation (4), with K = constant)

(4) $$\frac{\mathrm{df}}{\mathrm{dt}}=K\left(1-\frac{f}{a}\right)$$

Equation (3) can be written as follows (Equation (5)):

(5) $$\frac{df}{dt}=\tilde{K}\;\left(1-\frac{f}{a}\right)$$

where $\tilde{K}=\frac{a}{b}\;\left(1-\frac{f}{a}\right)$. In other terms, the dispersion kinetic can be represented by a Noyes–Whitney type equation (which corresponds to a first order kinetic) with a diffusion coefficient that is not constant but depends on the fraction of dispersed wax. The dependence of the diffusion coefficient on the concentration has been observed in other frameworks as, for example, diffusion of lipid in supercritical carbon dioxide^[^ ^30^ ^]^ and, more generally, in sorption and reaction‐diffusion systems that are characterized by a non‐Fickian diffusion.^[^ ^31^ ^]^ In the present framework it can be due to the particular geometry of the DESs.

The experimental dispersion curves for DES 8 and DES 6 (turbidimetric and gravimetric) exhibited a second order kinetics, well described by a generalized Hill‐type kinetic model with a shape parameter *c*  =  1, indicative of a non‐autocatalytic process. The corresponding regression model provided high‐quality fits with coefficients of determination consistently exceeding 0.97. Statistical metrics (AIC, BIC, F‐statistic, AICc) supported this model over the more general form with variable shape parameter. Although the generalized Hill function, a three parameter regression model, allowed for the inclusion of autocatalytic contributions (see the last paragraph), the fitted shape values were consistently close to 1, and the added complexity did not significantly improve fit quality.

In our case, the quadratic dependence of the simplified model (*c* = 1) captured the essential behavior, where the rate declines as saturation is approached. Mechanistically, dispersion likely proceeds via a two‐step interaction: partial solvation of surface wax, followed by full detachment as the molecule becomes solvated. The reduction in available DES molecules during the second phase accounts for the slowing dispersion rate, a behavior paralleled in other structured systems, including the diffusion of CO₂ in DESs^[^ ^55^ ^]^ and the solubilization of apolar compounds in eutectic media.^[^ ^56^ ^]^ Among the tested systems, DES 8 achieved the highest performance, as confirmed by RED analysis, dispersion mass, and turbidity increase. DES 6 showed intermediate behavior, while DES 12 demonstrated minimal dispersion. The convergence of multiple techniques (gravimetry, turbidimetry, profilometry) reinforced these conclusions. The RED model proved effective in prescreening solvent‐solute compatibility. However, it does not fully capture kinetic aspects or diffusion limitations, which must be experimentally confirmed. This aligns with recent perspectives emphasizing the need for kinetic modeling to complement thermodynamic descriptors in DES‐based systems.^[^ ^57^ ^]^ The results suggest that structured solvents like DESs exhibit concentration‐dependent kinetics, deviating from classical Fickian behavior. This supports the growing body of literature describing nonideal diffusion and phase interactions in DES‐based formulations.^[^ ^56^ ^]^

Finally, it should be noted that Figure 4 hints slightly at a sigmoidal profile (see the curves in the interval 0–60 min) in the case of turbidimetric measurements, which is not appreciable in the case of gravimetric measurements. This suggested to confront experimental data with the generalized Hill regression model which, in the case c≠1 assumes a sigmoidal profile (Equation (6)):

(6) $$\frac{f\left(t\right)}{a}=1-\frac{1}{1+{\left(\frac{t}{b}\right)}^{c}}$$

As already mentioned, the parameter estimation gave results close to *c*  =  1, in particular for gravimetric measurement where the estimation error is very small. Moreover, the comparison of the *c*  =  1 fitting with the c≠1 fitting is slightly in favor of the former.

Equation (6) is the solution of the following differential equation (Equation (7)):

(7) $$\frac{df}{dt}=kaf^{1-\frac{1}{c}}{\left(1-\frac{f}{a}\right)}^{1+\frac{1}{c}},b=\frac{c}{k}$$

where the term $f^{1-\frac{1}{c}}$ takes into account autocatalytic phenomena giving rise to the sigmoidal shape. Equation (7) reduces to Equation (3) when *c*  =  1 and to the logistic equation when c→∞. The sigmoidal behavior appears when c≠1 indicating that dispersion depends on both solubility potential and the already dispersed fraction. Although the present analysis suggested that autocatalytic phenomena are negligible (c ∼1), further investigations are required in order to get more insight on their possible role in the dispersion process. However, limitations of sensitivity in current techniques highlight the need for alternative strategies, such as microcalorimetry or spectroscopic monitoring, to explore initial dispersion stages.

## 3 Conclusion

This study explored the dispersion behavior of beeswax in a set of hydrophobic natural deep eutectic solvents (hNADESs), combining theoretical compatibility models with experimental kinetics. The primary goal was to evaluate the effectiveness of selected hNADESs in dissolving a nonpolar wax under mild conditions and to model the dispersion process through a simplified Hill‐type equation.

The results demonstrated that DES 8, based on dodecanoic and decanoic acid, exhibited the highest dispersion capacity, supported by all analytical methods used—turbidimetry, gravimetry, and wax height reduction. The applied kinetic model provided an accurate fit to the experimental data and enabled a quantitative interpretation of the dispersion mechanism, highlighting its nonlinear, saturation‐limited nature. These findings reinforce the potential of hNADESs as effective and sustainable alternatives to conventional solvents in conservation science and beyond. The proposed mathematical approach offers a generalizable tool for the evaluation of dispersion processes in structured solvents such as DES.

The results contribute to a deeper understanding of DES–wax interactions and offer a reproducible, quantitative approach for evaluating the performance of green solvents in the removal of hydrophobic materials from heritage surfaces.

## Conflict of Interests

The authors declare no conflict of interest.

## Data Availability

The data that support the findings of this study are available from the corresponding author upon reasonable request.
